# Galectin-1 in myelin repair

**DOI:** 10.18632/oncotarget.13455

**Published:** 2016-11-18

**Authors:** Mariana Rinaldi, Laura Thomas, Laura A. Pasquini

**Affiliations:** Department of Biological Chemistry, School of Pharmacy and Biochemistry, Institute of Biological Chemistry and Physics (IQUIFIB), University of Buenos Aires and National Research Council (CONICET), Argentina

**Keywords:** galectin-1, demyelination-remyelination, microghlia, lysolecithin, phagocytosis, Neuroscience

Galectin-1 (Gal-1) is a member of a highly conserved family of animal lectins which binds to the common disaccharide [Galβ(1-4)-GlcNAc] on both *N*- and *O*-glycans decorating cell surface glycoconjugates. Current evidence supports a role for Gal-1 in the pathophysiology of multiple sclerosis (MS), one of the most prevalent chronic inflammatory diseases, as approximately one third of MS patients generate high titres of anti-Gal-1 antibodies. Four different lesion types have been described in MS: pattern-1 and -2 lesions are thought to be mediated by the autoimmune response, while pattern-3 and -4 lesions are considered primary oligodendropathy. The first two types are experimentally simulated in mice by experimental autoimmune encephalomyelitis (EAE), while the second two are mimicked by toxic models such as cuprizone (CPZ) or lysolecithin (LPC) administration. Studies in EAE models have demonstrated that Gal-1 is highly expressed in the acute phase of the disease and that its deficiency leads to severe inflammation-induced neurodegeneration [1]. Regarding its mechanism of action, Gal-1 binds to core 2 *O*-glycans on CD45 and induces its retention on the microglial surface, increasing CD45 phosphatase activity and inhibitory function. Adoptive transfer of Gal-1-secreting astrocytes or administration of recombinant Gal-1 at disease onset ‘after inflammatory cells have entered the CNS’ attenuates EAE severity and reduces microglial activation [1]. However, these studies using the EAE model, in which damage is the consequence of an immunological attack to myelin, have failed in determining whether Gal-1 effects are due to damage suppression or response modulation against demyelination.

**Figure 1:**
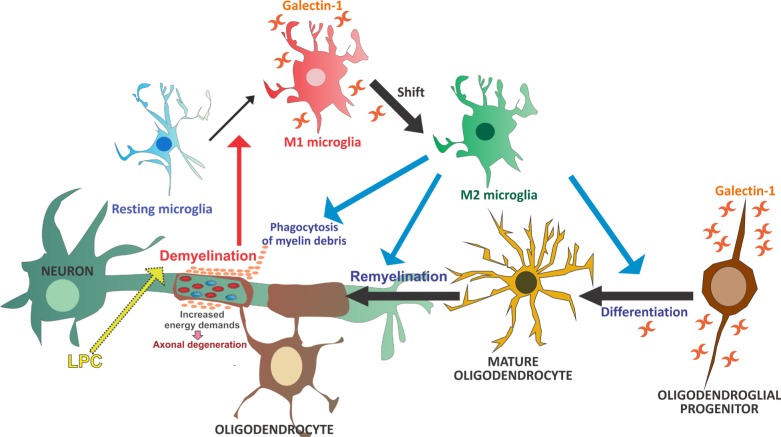
Resting microglia are activated toward an M1 phenotype in response to LCP-induced demyelination Gal- 1 treatment targets these M1-activated microglia, promotes their shift toward an M2-polarized phenotype, thus increasing their myelin phagocytic capacity, and leads to oligodendroglial differentiation and remyelination.

On the other hand, Rinaldi et al. [2] have shown that, upon focal LPC-induced demyelination, Gal- 1 treatment induces both a significant decrease in demyelinated areas and more efficient remyelination, as observed when Gal-1 is administered immediately after or 4 days after LPC, respectively. This effect is accompanied by an attenuated oligodendroglial progenitor, microglial and astroglial response, which also reflects lesser myelin damage. This effect is mediated by Gal-1 targeting activated M1 microglia ‘inflammatory and degenerative’, which accelerates the shift toward an M2 phenotype ‘antiinflammatory and regenerative’ and increases myelin phagocytic capacity, which in turn circumvents damage. This switch from M1 to M2 polarization takes place 10 days after LPC injection in non-treated animals, in agreement with previous studies [3], but as early as 3 days after LPC injection in Gal-1-treated ones. These Gal-1-induced changes are essential to the onset of remyelination, as myelin debris has been proven to inhibit oligodendroglial progenitor cell differentiation [4]. Previous evidence shows oligodendrocyte differentiation to be enhanced *in vitro* with M2 cell-conditioned media and impaired *in vivo* following intra-lesional M2 cell depletion [3]. In agreement with *in vivo* results, *in vitro* studies show that Gal-1 targets activated microglia, promoting an increase in their myelin phagocytic capacity and their shift toward an M2 phenotype, and leads to oligodendroglial differentiation. However, these results also show that a Gal-1-induced shift toward an M2 phenotype cannot single-handedly induce factor release to enhance oligodendroglial maturation. Instead, a direct effect of Gal-1 on oligodendroglial survival and/or differentiation appears to be required, together with microglial-oligodendrocyte contact enabling the phagocytosis of myelin to favor remyelination (Figure 1). Taken together, this body of evidence supports the use of Gal-1 as a potential therapeutic agent for demyelinating diseases such as MS [2].

Axonal injury occurs early in acute MS lesions. Our group has also recently shown that dimeric Gal-1 prevents neuroregeneration-blocking signals triggered by Sema3A binding to the NRP-1/PlexinA4 complex in spinal cord injury, which may pave the way for the use of this lectin in the treatment of pathologies involving neurodegeneration [5]. Rinaldi's studies further show that axonal degeneration and LPC-induced loss of contact between axon and myelin sheath are reversed by Gal-1 treatment. This neuroprotective effect of Gal- 1 has also been established in studies using electronic microscopy, which showed more efficient myelination and remyelination in the presence of Gal-1, reflected by an increase in the levels of myelin deposition, the number of myelinated axons and the number of myelin turns, and a decrease in g-ratio and axonal mitochondrial area [2].

*In vitro* studies have identified Sema3A as a powerful, selective and reversible inhibitor of oligodendroglial precursor cell differentiation, while *in vivo* analyses have shown Sema3A administration to induce demyelination and hinder remyelination in LPC-treated rats. Moreover, active MS lesions have provided evidence of Sema3A expression blocking OLG differentiation [6]. In addition, recently published work has shown microglial NRP-1 ablation to block microglial signaling and antiinflammatory M2 polarization [7]. In other words, Gal-1 beneficial effects on remyelination could also be thought to involve either the blocking of Sema3A binding to NRP-1/PlexinA4 in OLG, or its interaction with NRP-1 in microglial cells, which might favor a shift toward an M2 phenotype. These hypotheses are currently being evaluated by our group.

To sum up, increasing evidence demonstrates Gal-1 participation in glial response modulation [1, 2, 8]. In particular, the T cell-independent LPC-induced demyelination model used by Rinaldi et al. [2] makes it possible to discriminate the effects of Gal-1 on the glial response from its possible effects on damage suppression, as damage occurs at the time of LPC injection and is only followed by the regenerative process. These findings highlight the therapeutic efficacy of Gal-1 in the recovery of pattern-3 and -4 lesions, characterized by primary oligodendropathy [2]. Altogether, studies so far support the use of Gal-1 for the treatment of CNS demyelinating diseases such as MS, through microglial modulation, polarization and phagocytosis, and remyelination.
